# Pablo Picasso (1881–1973). Guernica (1937). Oil on canvas, 350 cm x 782 cm

**DOI:** 10.3201/eid0906.AC0906

**Published:** 2003-06

**Authors:** Polyxeni Potter

**Affiliations:** *Centers for Disease Control and Prevention, Atlanta, Georgia, USA

**Figure:**
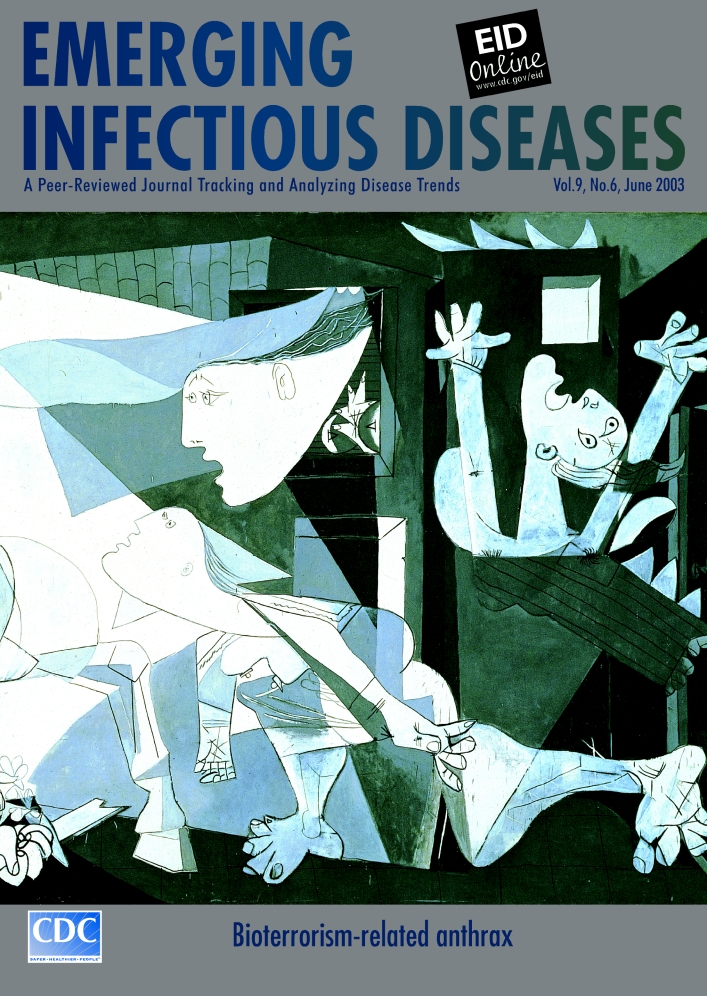
Pablo Picasso (1881–1973). Guernica (1937). Oil on canvas, 350 cm x 782 cm Copyright ARS, NY. Museo Nacional Centro de Arte Reina Sofía, Madrid, Spain. Photo by John Bigelow Taylor/Art Resource, NY

“It isn’t up to the painter to define the symbols,” said Pablo Picasso when asked to explain his celebrated mural, Guernica. “….The public who look at the picture must interpret the symbols as they understand them.” Picasso himself did not know what the work would turn out to be when he was commissioned to paint the centerpiece for the Spanish Pavilion of the 1937 World’s Fair in Paris: “A painting is not thought out in advance. While it is being done, it changes as one’s thoughts change. And when it’s finished, it goes on changing, according to the state of mind of whoever is looking at it” (*1*,*2*).

The official theme of the World’s Fair was modern technology, but the inspiration for Picasso’s mural came from world events—specifically the Nazi bombing and virtual obliteration of Guernica, an ancient Basque town in northern Spain. The bombardment of this nonmilitary target took little more than 3 hours, during which airplanes, plunging low from above the center of town, machine-gunned the townspeople who had taken refuge in the fields (*3*). News reports and horrific photographs of the massacre quickly reached Paris and provided the story line for perhaps the best-known painting of the 20th century. Started 6 days after the bombing and completed in 5 weeks, the monumental mural captured the agony brought on by brutality and violence.

A native of Andalusia, Spain, who lived most of his life in France, Picasso was the most innovative artist of his era and perhaps any era. His complex genius is usually tracked in a series of overlapping periods beginning in 1901. A master of classical art, he painted the poor, whose ordinary activities he imbued with melancholy and lyricism (blue period, rose period). Around 1905, influenced by Cézanne and African sculpture, he experimented with fragmented and distorted images and became one of the founders of modern abstraction (literally “the drawing away from or separating”).

Destroying in order to create, Picasso dismantled traditional forms and sought the inner geometry of objects and the human figure. His Les Demoiselles d’Avignon (1907) marked the beginning of analytic cubism, the harsh intellectual style (also of Braque and Gris) in which decomposition of objects into geometric lines and contours is carried to an extreme (*5*). Figures and their surroundings are broken into “angular wedges or facets,” shaded to appear three-dimensional. We cannot tell if the fragments are concave or convex; some seem “chunks of modified space,” others translucent bodies comprising a fantastic world of compounded voids and solids (*4*).

Traditional art confines its subject to one time and place. Cubism allows the artist to express what Albert Einstein defined in 1905 in his theory of relativity: a new sense of time, space, and energy in which moving figures become an extension of the environment from which they are indistinguishable (*4*). As art, the world, and self converge, continuity and brokenness, symmetrical progression, life and death, pain and hope can be viewed within a broader aesthetic reality (*6*). Around 1909, Picasso eliminated color, replacing it with a range of gray and brown tones to which he added new elements, paper cutouts, numbers, and letters, creating collages and other new techniques that further separated the work of art from any representation of reality. In a later form, cubism became “synthetic,” more representational and flat, and included bright decorative patterns (as in The Three Musicians, 1921).

“Art is the lie that tells the truth,” Picasso once said, articulating how an abstract painting could pack so much passion. Guernica does not represent the event that inspired it. Rather, in a series of allegorical images, it evokes the complexity and depth of suffering caused by the event. In a systematically crowded composition (deliberately undermining the academic rules of art), figures are crammed into the foreground: screaming mother cradling dead child, corpse with wide open eyes, arm holding lamp, fighter’s arm with weapon, menacing human-faced bull, gored horse. In open darkness and surrounded by burning buildings, the figures seem united in a sublime lament, reminiscent of Mediterranean funeral rites (*5*). Their symbolism defies exact interpretation; they owe their terrific eloquence to “what they are, not what they mean.” With flawless internal logic, the anatomical dislocations, fragmentations, and transformations expose the stark reality of unbearable pain (*4*).

As if conceived during a lightening strike, Guernica shocks in near monochrome, exemplifying the triumph of pure abstracted form. Its symbolism, punctuated by innovating techniques (e.g., inclusion of newsprint), transforms a local event to a universal icon of terror in the aftermath of violence. The figures, human and not, in primitive flight, embody the horror of living creatures under attack. With undeniable clarity, Picasso spells out in modern terms humanity’s condemnation of unnecessary suffering, the agony caused not by unavoidable disasters or indecipherable diseases but by unimaginable intentional violence, such as witnessed in the deliberate release of biological agents.
